# Association between lipid biomarkers and osteoporosis: a cross-sectional study

**DOI:** 10.1186/s12891-021-04643-5

**Published:** 2021-09-06

**Authors:** Bo Kan, Qianqian Zhao, Lijuan Wang, Shanshan Xue, Hanqing Cai, Shuman Yang

**Affiliations:** 1 Department of Clinical Laboratory, The Second Hospital of Jilin University, Jilin Changchun, China; 2 Department of Epidemiology and Biostatistics, School of Public Health, Jilin University, 232-1163 Xinmin Street, Jilin 130021 Changchun, China; 3 Department of Endocrine, The Second Hospital of Jilin University, Jilin Changchun, China

**Keywords:** Lipid biomarkers, Osteoporosis, Bone mineral density (BMD), Risk assessment

## Abstract

**Background:**

Osteoporosis and cardiovascular diseases (CVDs) are 2 major public health problems. Osteoporosis and CVDs may be linked but the association between lipid profile and osteoporosis is still controversial. The purpose of this study was to examine the associations of total cholesterol (TC), low-density lipoprotein cholesterol (LDL-C), high-density lipoprotein cholesterol (HDL-C) and triglyceride (TG) with osteoporosis.

**Methods:**

Using inpatients’ and outpatients’ electronic medical records (EMR) and dual X-ray absorptiometry (DXA) database stored at The Second Hospital of Jilin University, we included 481 individuals with complete and valid lipid and bone mineral density (BMD) data in 2017. Serum samples were used to measure TC, LDL-C, HDL-C and TG. Femoral neck and total hip BMD were measured by DXA; osteoporosis was defined as femoral neck or total hip T-score ≤ -2.5. Multivariable logistic regression models were used to test the associations of TC, LDL-C, HDL-C and TG with osteoporosis.

**Results:**

The mean age for included individuals was 62.7 years (SD = 8.6 years); 60.1 % of them were female. Each standard deviation (SD) increase in TC (Odds Ratio [OR]: 1.48; 95 % Confidence Interval [CI]: 1.06–2.07) and TG (OR: 1.67; 95 % CI: 1.16–2.39) were associated with increased risk of osteoporosis; LDL-C and HDL-C levels were not associated with osteoporosis. Age, sex and body mass index (BMI) did not interact with the relationships of TC and TG with osteoporosis (all *P* > 0.10).

**Conclusions:**

Higher TC and TG levels were associated with greater risk of osteoporosis in this cross-sectional study.

**Supplementary Information:**

The online version contains supplementary material available at 10.1186/s12891-021-04643-5.

## Background

Worldwide, osteoporosis and cardiovascular diseases (CVDs) are 2 major public health problems [1, 2]. Up to 49 million individuals have osteoporosis in North America, Europe, Japan, and Australia [3]. In 2017, CVDs lead to approximately 17.8 million deaths worldwide [4, 5]. Osteoporosis and CVDs both significantly increase risk of morbidity and mortality [6, 7].

Osteoporosis and CVDs may be linked [8, 9]. Osteoporosis and CVDs share many risk factors, such as advancing age, premature menopause, genetics, a sedentary lifestyle and smoking, etc. [10, 11]. There is evidence suggesting that individuals with CVDs have 1.69 folds higher risk of osteoporosis than those without CVDs [12]. In addition, osteoporotic patients are 1.2 to 1.4 times more susceptible to cardiovascular events than those without osteoporosis [10, 13].

Lipid profile is useful for assessing risk of CVDs [14]. There is also *vitro* evidence suggesting lipids are involved in the development of osteoporosis [15].However, the association between lipid profile and osteoporosis in human studies is still controversial [16–25]. Majority of human studies suggested that there were negative associations between lipid biomarkers and bone mineral density (BMD) [16, 17, 19, 22–25]. However, Ersoy et al. [18] and Lahon et al. [20] found that low-density lipoprotein cholesterol (LDL-C) was positively correlated with BMD. Ghadiri-Anari et al. [21] suggested a null association between lipids and BMD. Because of the conflicting associations of lipids with BMD and/or osteoporosis, we examined the associations of total cholesterol (TC), LDL-C, high-density lipoprotein cholesterol (HDL-C) and triglyceride (TG) with osteoporosis in a Chinese hospital-based population.

## Methods

### Study setting and subjects

The data were drawn from inpatients’ and outpatients’ electronic medical records (EMR) and dual X-ray absorptiometry (DXA) database stored at The Second Hospital of Jilin University, which is a major diagnosis and treatment center for severe diseases (i.e., cancers and CVDs) in northeast China. The linked for EMR and DXA databases capture inpatients’ and outpatients’ demographics (i.e., age and sex), anthropometry data (e.g., body height and weight), clinical laboratory measurement records, prescription drug dispensation records, clinical diagnoses and BMD data.

We identified individuals with complete and valid data on femoral neck and total hip BMD, TC, LDL-C, HDL-C and TG in 2017 in this study. We excluded individuals: (1) with age < 50 years old at BMD test; (2) with early (< 40 years) menopause; (3) with a lipid-lowering therapy, synthyroid or hormone-replacement therapy; (4) with osteoporosis-related diseases such as cancer, thyroid disease, hypopituitarism, rheumatoid arthritis, chronic renal failure or renal dysfunction (creatinine > 442 µmol/L), chronic liver disease or liver dysfunction (aspartate aminotransferase or alanine aminotransferase > 80 U/L); (5) with a surgical history of bilateral salpingo-oophorectomy menopause or a history of bone surgery at the lumbar spine or hip. There are no data on osteoporosis medications in our study. Osteoporosis medications are prescribed after the patients are diagnosed with osteoporosis through DXA testing. To partially excluding the individuals taking anti-osteoporosis medication, we only include individuals at their first DXA tests in our study. This study was approved by The Second Hospital of Jilin University Life Science Ethics Committee (2017 Research Approval No.13).

### BMD measurement

Femoral neck and total hip BMD were measured by a DXA fan-beam bone densitometer (Discovery Wi, Hologic, Bedford, MA, USA). The control spine phantom scan performed each day had a long-term (more than 5 years) coefficient of variation (CV) of < 0.5 %. A BMD T-score was calculated using the following formula:

$$\mathrm T=\left(\mathrm X-\mathrm\mu^{\mathrm{reference}}\right)/\mathrm{SD}^{\mathrm{reference}}$$

Where X = the observed BMD of the patients, µ^reference^ = BMD in young adults, and SD^reference^ = standard deviation of BMD in young adults.

We defined osteoporosis as femoral neck or total hip T-score ≤ -2.5 [26]; the reference for calculating T-score was based on BMD data from Whites 20–29 years old in the third National Health and Nutrition Examination Survey (NHANES III) [27].

### Blood draw and storage

Fasting blood samples (≥ 8 h) were drawn from individuals with spray-coated silica and a polymer gel evacuated sterile collection tubes (BD, Becton, Dickinson and Company, Franklin Lakes, New Jersey, USA) by nurses; these blood samples were centrifuged to obtain serum at clinical laboratory department.

### Lipid biomarkers ascertainment

Within 2 h following blood draw, serum samples were used to directly measure TC, LDL-C, HDL-C and TG; their levels were determined by a biochemical analyzer (7600 model, Hitachi, Tokyo, Japan). The CVs for TC, LDL-C, HDL-C and TG measurements were 2.5 %, 3.1 %, 5.1 and 1.9 %, respectively.

### Covariate ascertainment

The covariates were extracted from EMR and DXA databases. The covariates for this study included age, sex, body mass index (BMI), disease diagnoses (e.g., type 2 diabetes, hypertension, ischemic heart disease, ischemic stroke, osteoarthritis), diabetes complications (e.g., neuropathy, retinopathy, nephropathy), diabetes medications (e.g., insulin, biguanide, sulfonylureas, others), antihypertension medications (e.g., calcium channel blockers, angiotensin 2 receptor blockers, beta-blockers, diuretics, angiotensin converting enzyme inhibitors), liver function biomarkers, bone biochemical markers (e.g., alkaline phosphatase, calcium and phosphorus), thyroid function biomarkers and glucose metabolic biomarkers. The covariates included in the analysis could be potential confounders or effect modifiers that maybe associated with lipid biomarkers and femoral neck or total hip BMD [16, 22, 24]. Height and weight were measured using a wall-mounted stadiometer (to the nearest 0.1 cm) and an electronic scale (to the nearest 0.1 kg). BMI was calculated as weight (kilograms) divided by the square of the height (meters). Any past use of diabetes medications and antihypertension medications were self-reported. Disease diagnoses were either self-reported and/or clinically diagnosed based on specific criteria. The biomarkers were measured using established methods in the clinical setting [28]. To further examine whether menopausal age had an impact on the association between lipid biomarkers and osteoporosis, we also considered self-reported menopausal age in women.

### Statistical analysis

Multivariable logistic regression models were used to test the associations of TC (per SD increase), LDL-C (per SD increase), HDL-C (per SD increase) and TG (per SD increase) with osteoporosis. Due to the high correlations between lipid biomarkers (Pearson r up to 0.70), we included each lipid biomarker in one model. Models were further adjusted for sex, age, BMI, type 2 diabetes, neuropathy, biguanide, calcium channel blockers, angiotensin converting enzyme inhibitors, alanine transaminase, albumin, total bilirubin and alkaline phosphatase; variables considered but not included in the adjusted model were hypertension, ischemic heart disease, ischemic stroke, osteoarthritis, retinopathy, nephropathy, insulin, sulfonylureas, others, angiotensin 2 receptor blockers, beta-blockers, diuretics, aspartate aminotransferase, total protein, gamma-glutamyltransferase, calcium, phosphorus, free thyroxine 4, thyroid-stimulating hormone, glycated hemoglobin A1c and fasting glucose, because they failed to meet *P* < 0.10 criteria under bivariate analyses with osteoporosis. Because total bilirubin and direct bilirubin are highly associated (*r* = 0.80), to avoid the effect of collinearity, direct bilirubin was not included in the multivariable logistic regression models.

Multivariable linear regression models were used to test the associations between lipid biomarkers and femoral neck or total hip BMD. Variables included in the model were the same as above.

TC, LDL-C, HDL-C and TG were classified as normal and abnormal groups according to their clinical reference values. In serum, TC ≥ 5.20 mmol/L and/or LDL-C ≥ 3.40 mmol/L and/or HDL-C < 1.00 mmol/L and/or TG ≥ 1.70 mmol/L were defined as abnormal groups [29]. Multivariable logistic regression models were used to test the associations of each lipid biomarker group with osteoporosis. Again, adjusted covariates were the same as above.

Multivariable logistic regression models were used to test the associations of each lipid biomarker group with osteoporosis in women only and in individuals with any chronic disorders. In the subgroup analysis of women only, we further adjusted menopausal age in addition to the covariates as mentioned above.

All analyses were conducted with SPSS (version 24.0, IBM, Inc., New York, USA).

## Results

**Fig. 1:**
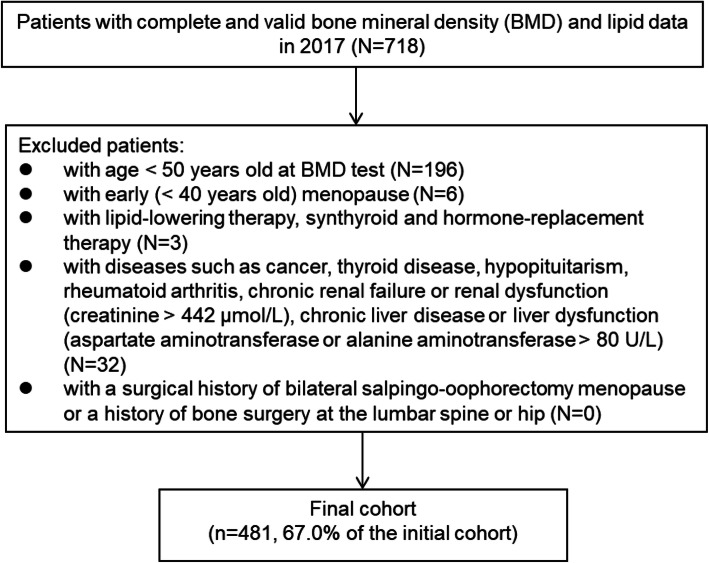
Flow chart for cohort inclusion and exclusions

After excluding ineligible individuals (Fig. 1), we included 481 individuals for this study. As compared to excluded individuals, included individuals were older (62.7 ± 8.6 vs. 45.2 ± 10.1 years; *P* < 0.001) and more likely to be female (60.1 vs. 49.0 %; *P* < 0.001); BMI was not significantly different between included and excluded individuals (25.7 ± 3.5 vs. 25.7 ± 4.3 kg/m^2^; *P* = 0.886). We only had menopausal age for 234 women (81.0 % of all women included in this study). Based on the women with complete data on menopausal age, we found that women with osteoporosis had younger menopausal age than those without osteoporosis (47.7 ± 3.5 vs. 49.4 ± 3.2 years; *P* < 0.001).

Among included individuals, 357 (74.2 %), 228 (47.4 %) and 84 (17.5 %) individuals had type 2 diabetes, hypertension and ischemic heart disease diagnoses, respectively. The mean age for included individuals was 62.7 years (SD = 8.6 years); 60.1 % of individuals were female; the average BMI for included individuals was 25.7 kg/m^2^ (SD = 3.5 kg/m^2^). As shown in Table 1, osteoporotic individuals tended to be older, more likely to be female and had higher proportion of calcium channel blockers medication, angiotensin converting enzyme inhibitors medication, higher levels of TC, alkaline phosphatase and a lower proportion of type 2 diabetes, biguanide medication, lower levels of BMI, alanine transaminase, albumin and total bilirubin.

**Table 1:** Characteristics of study participants by osteoporosis status

Variable	No Osteoporosis (*n* = 411)	Osteoporosis (*n* = 70)	*P*
**Demographics**			
Female (n, %)^b^	224 (54.5)	65 (92.9)	< 0.001
Age (years)	61.5 (8.0)	70.1 (8.4)	< 0.001
**Anthropometry**			
Height (cm)	162.8 (8.6)	153.7 (5.8)	< 0.001
Weight (kg)	68.9 (11.4)	57.1 (9.3)	< 0.001
Body mass index (kg/m^2^)	26.0 (3.4)	24.2 (3.8)	< 0.001
**Disease diagnosis (n, %)**^b^			
Type 2 diabetes	317 (76.39)	40 (60.61)	0.007
Hypertension	198 (47.71)	30 (45.45)	0.733
Ischemic heart disease	70 (16.87)	14 (21.21)	0.388
Ischemic stroke	10 (2.41)	3 (4.55)	0.320
Osteoarthritis	6 (1.45)	1 (1.52)	0.965
**Diabetes complication (n, %)**^b^			
Neuropathy	199 (48.42)	26 (37.14)	0.082
Retinopathy	104 (25.30)	16 (22.86)	0.662
Nephropathy	7 (1.69)	2 (3.03)	0.454
**Diabetes medication (n, %)**^b^			
Insulin	178 (43.3)	28 (40.0)	0.605
Biguanide	75 (18.3)	5 (7.1)	0.027
Sulfonylureas	27 (6.6)	6 (8.6)	0.541
Others	22 (5.4)	5 (7.1)	0.548
**Antihypertension medication (n, %)**^b^			
Calcium channel blockers	34 (8.27)	11 (15.71)	0.048
Angiotensin 2 receptor blockers	22 (5.35)	3 (4.29)	0.710
Beta-blockers	9 (2.19)	2 (2.86)	0.730
Diuretics	6 (1.46)	1 (1.43)	0.984
Angiotensin converting enzyme inhibitors	2 (0.49)	2 (2.86)	0.044
**Lipid biomarker**			
Total cholesterol (mmol/L)	5.06 (1.26)	5.42 (1.35)	0.028
Low-density lipoprotein cholesterol (mmol/L)	2.85 (0.86)	3.04 (0.99)	0.101
High-density lipoprotein cholesterol (mmol/L)	1.14 (0.31)	1.19 (0.31)	0.235
Triglyceride (mmol/L)^a^	1.57 (1.12, 2.44)	1.83 (1.27, 3.05)	0.189
**Liver function biomarker**			
Alanine transaminase (U/L)^a^	21.00(15.00, 30.00)	16.50(11.00, 22.00)	0.008
Aspartate aminotransferase (U/L)^a^	18.00(15.00, 23.00)	17.00(15.00, 22.00)	0.420
Total protein (g/L)	71.35 (6.47)	70.32 (8.17)	0.242
Albumin (g/L)	43.10 (4.21)	41.12 (5.32)	< 0.001
Total bilirubin (µmol/L)	13.10 (5.54)	11.51 (5.15)	0.027
Direct bilirubin (µmol/L)^a^	3.25 (2.27, 4.49)	2.60 (1.90,3.99)	0.092
Gamma-glutamyltransferase (U/L)^a^	27.00 (20.00, 43.00)	20.00 (15.00, 33.00)	0.452
**Bone biochemical marker**			
Alkaline phosphatase (U/L)	89.75 (28.98)	108.20 (51.70)	< 0.001
Calcium (mmol/L)	2.34 (0.13)	2.31 (0.15)	0.170
Phosphorus (mmol/L)	1.13 (0.20)	1.12 (0.21)	0.625
**Thyroid function biomarker**			
Free thyroxine 4 (Ft4)(pmol/L)	15.94 (3.15)	16.36 (11.45)	0.575
Thyroid-stimulating hormone (TSH) (mIU/L)^a^	2.03 (1.23, 3.11)	1.98 (0.96, 3.74)	0.881
**Glucose metabolic biomarker**			
Glycated hemoglobin A1c (%)	8.96 (2.10)	8.45 (2.25)	0.112
Fasting glucose (mmol/L)	9.20 (3.76)	8.63 (5.04)	0.301

Continuous variables with normal distribution are shown as means (standard deviations)

^a^continuous variables with skewed distribution are shown as medians (inter-quartile ranges)

^b^categorical variables are shown as frequencies (%)

As shown in Table 2, among all 481 individuals, in the adjusted model, each SD increase in TC (Odds Ratio [OR]: 1.48; 95 % Confidence Interval [CI]: 1.06–2.07) and TG (OR: 1.67; 95 % CI: 1.16–2.39) were associated with increased risk of osteoporosis; LDL-C and HDL-C levels were not associated with osteoporosis. Age, sex and BMI did notmodify the relationships of TC and TG with osteoporosis (all *P* > 0.10). When T-score of BMD was treated as continuous variable, we observed similar trends of associations of TC and TG with BMD T-score though these associations did not reach statistical significance (Table S1). After classifying lipid biomarkers according to their clinical cut-off values, we found abnormal higher TG levels in serum were associated with higher risk of osteoporosis (Table S2). In women, after further adjusting menopausal age, we found that TG was still significantly associated with osteoporosis (OR: 1.98; 95 % CI: 1.24–3.17; Table S3). In the subgroup analysis of individuals with any chronic disorders, we also found elevated TC and TG levels were associated with higher risk of osteoporosis (Table S4).

**Table 2:** Adjusted^a^ odds ratio (ORs) and 95 % confidence intervals (95 % CIs) for osteoporosis associated with lipid biomarkers (per standard deviation increase)

Independent Variable	*P*	OR (95 %CI)
Total cholesterol	0.022	1.48 (1.06–2.07)
Low-density Lipoprotein cholesterol	0.108	1.29 (0.95–1.76)
High-density Lipoprotein cholesterol	0.547	1.11 (0.79–1.56)
Triglyceride	0.005	1.67 (1.16–2.39)

^a^Adjusted for sex, age, body mass index, type 2 diabetes, neuropathy, biguanide, calcium channel blockers, angiotensin converting enzyme inhibitors, alanine transaminase, albumin, total bilirubin and alkaline phosphatase

## Discussion

In this study of individuals aged ≥ 50 years, we demonstrated that higher TC and TG levels were associated with greater risk of osteoporosis. Serum LDL-C and HDL-C levels were not associated with osteoporosis.

The positive relationships of TC and TG levels with osteoporosis in our study are consistent with some previous studies [19, 23, 25, 30], in which there were positive correlations of TC and TG levels with osteoporosis. We found no significant associations of HDL-C and LDL-C with osteoporosis. These results are also in line with the findings reported by Ghadiri-Anari A et al. [21], those found insignificant associations between lipids and BMD. However, existing epidemiological studies suggested that there are still conflicting associations of HDL-C and LDL-C with osteoporosis [17, 18, 20]. These conflicting results are likely attributed to the different study sample sizes, study populations and covariates adjusted in the study. For example, the insignificant association between LDL-C and osteoporosis in our study may be a false negative finding due to the limited sample size. In addition, numerous studies suggested that different study populations may lead to different results [16, 22, 23]. Majority of included individuals (82.5 %) in our study had chronic diseases (i.e., type 2 diabetes, hypertension and ischemic heart disease). These diseases were suggested to be related to lipids and osteoporosis [31–33]. However, most of the studies on the association between lipids and osteoporosis were based on generally healthy populations [22, 25, 34]. This may lead to conflicting results. Lastly, as compared to previous studies [16, 21, 24], they used different covariates to adjusting the association between lipids and osteoporosis. This may also cause inconsistent results between studies.

Regardless of consistent or conflicting findings, the positive associations of TC and TG levels with BMD can be explained by several biological mechanisms. First, the nuclear hormone receptor peroxisome proliferator activated receptor γ (PPAR γ) may play a role in the relationship of lipid biomarkers and BMD. PPAR γ can be activated by lipid metabolites. When PPAR γ level increases, osteogenesis is inhibited [35]; this leads to increased bone loss. Second, higher lipid levels are associated with an increase in oxidized lipids and higher oxidative stress levels. Higher oxidative stress level could inhibit osteoblast differentiation as well as to promote adipocyte differentiation [36, 37]. Third, higher serum TG levels are positively associated with higher bone marrow fat [38], which leads to lower trabecular BMD [39].

Our study has some strengths. DXA was used to diagnose osteoporosis. In addition, the lipid biomarkers (e.g., TC, LDL-C, HDL-C, TG) were measured using established methods in the clinical setting [40]. This ensured the reliability and accuracy of osteoporosis ascertainment and lipid measurements, which allowed us to analyzing the associations between lipids and osteoporosis with less bias. Several limitations for this study are acknowledged. First, the findings were derived from a cross-sectional study. Therefore, we cannot make causal inference about the association between lipid biomarkers and BMD. Second, we only analyzed some inpatients and outpatients in The Second Hospital of Jilin University. For example, 74.2 % of individuals in our study were type 2 diabetic patients, while the prevalence of type 2 diabetes in Jilin Province is 17.5 % [41]. Thus, the findings from this selected population may not be able to generalize to general population or the population in the hospital. Last, due to the absent data, we did not consider individuals’ lifestyle factors (i.e., smoking and alcohol use and physical activity) and vitamin D levels. However, in a previous study, adjustment of vitamin D levels did not appreciably change the associations of TC and TG with osteoporosis [42].

## Conclusions

In conclusion, there were associations between serum TC and TG and osteoporosis in this study. These findings extended our understanding about the lipid profile and BMD. High quality prospective studies on relationship between lipid biomarkers and BMD are still warranted to confirm or oppose our results.

## Supplementary Information

**Additional file 1: Table S1.** Multivariable linear regression analysis* of the associations between lipid biomarkers and T-score of femoral neck and total hip bone mineral density (BMD). **Table S2.** Adjusted* odds ratio (ORs) and 95% confidence intervals (95% CIs) for osteoporosis associated with abnormal lipid biomarkers. **Table S3.** Adjusted* odds ratio (ORs) and 95% confidence intervals (95% CIs) for osteoporosis in women associated with lipid biomarkers (per standard deviation increase). **Table S4.** Adjusted* odds ratio (ORs) and 95% confidence intervals (95% CIs) for osteoporosis associated with lipid biomarkers (per standard deviation increase) in patients with chronic disorders

## Data Availability

The datasets used and analysed during the current study are available from the corresponding author on reasonable request.

## Abbreviations

CVDs: Cardiovascular diseases
TC: Total cholesterol
LDL-C: Low-density lipoprotein cholesterol
HDL-C: High-density lipoprotein cholesterol
TG: Triglyceride
EMR: Electronic medical records
DXA: Dual X-ray absorptiometry
BMD: Bone mineral density
SD: Standard deviation
OR: Odds ratio
CI: Confidence interval
BMI: Body mass index
NHANES III: The third National Health and Nutrition Examination Survey
SDs: Standard deviations
PPARγ: Peroxisome proliferator activated receptor γ
