# Supplementary material for: Association between lipid biomarkers and osteoporosis: a cross-sectional study
Source: BMC Musculoskelet Disord. 2021 Sep 6;22:759. doi: 10.1186/s12891-021-04643-5 (PMC8419899; doi:10.1186/s12891-021-04643-5)
Supplement: Supplementary file 1 — Additional file 1: Table S1. Multivariable linear regression analysis* of the associations between lipid biomarkers and T-score of femoral neck and total hip bone mineral density (BMD). Table S2. Adjusted* odds ratio (ORs) and 95% confidence intervals (95% CIs) for osteoporosis associated with abnormal lipid biomarkers. Table S3. Adjusted* odds ratio (ORs) and 95% confidence intervals (95% CIs) for osteoporosis in women associated with lipid biomarkers (per standard deviation increase). Table S4. Adjusted* odds ratio (ORs) and 95% confidence intervals (95% CIs) for osteoporosis associated with lipid biomarkers (per standard deviation increase) in patients with chronic disorders [file 12891_2021_4643_MOESM1_ESM.docx]

**Additional file**

**Table S1 Multivariable linear regression analysis* of the associations between lipid biomarkers and T-score of femoral neck and total hip bone mineral density (BMD)**

| Independent Variable | *β* (*P*) | |
| --- | --- | --- |
|  | Femoral Neck BMD T-score | Total Hip BMD T-score |
| Total cholesterol | -0.060 (0.186) | -0.045 (0.333) |
| Low-density  Lipoprotein cholesterol | -0.029 (0.516) | -0.042 (0.357) |
| High-density  Lipoprotein cholesterol | -0.042 (0.367) | -0.035 (0.459) |
| Triglyceride | -0.070 (0.129) | -0.061 (0.196) |

*Adjusted for sex, age, body mass index, type 2 diabetes, neuropathy, biguanide, calcium channel blockers, angiotensin converting enzyme inhibitors, alanine transaminase, albumin, total bilirubin and alkaline phosphatase.

**Table S2 Adjusted* odds ratio (ORs) and 95% confidence intervals (95% CIs) for osteoporosis associated with abnormal lipid biomarkers**

| Independent Variable | *P* | OR (95%CI) |
| --- | --- | --- |
| Total cholesterol | 0.235 | 1.49 (0.77-2.90) |
| Low-density Lipoprotein cholesterol | 0.239 | 1.50 (0.77-2.93) |
| High-density Lipoprotein cholesterol | 0.696 | 0.84 (0.36-1.98) |
| Triglyceride | 0.010 | 2.46 (1.24-4.88) |

*Adjusted for sex, age, body mass index, type 2 diabetes, neuropathy, biguanide, calcium channel blockers, angiotensin converting enzyme inhibitors, alanine transaminase, albumin, total bilirubin and alkaline phosphatase.

**Table S3 Adjusted* odds ratio (ORs) and 95% confidence intervals (95% CIs) for osteoporosis in women associated with lipid biomarkers (per standard deviation increase)**

| Independent Variable | *P* | OR (95%CI) |
| --- | --- | --- |
| Total cholesterol | 0.061 | 1.46 (0.98-2.17) |
| Low-density Lipoprotein cholesterol | 0.255 | 1.22 (0.87-1.73) |
| High-density Lipoprotein cholesterol | 0.813 | 1.05 (0.70-1.57) |
| Triglyceride | 0.004 | 1.98 (1.24-3.17) |

*Adjusted for sex, age, menopausal age, body mass index, type 2 diabetes, neuropathy, biguanide, calcium channel blockers, angiotensin converting enzyme inhibitors, alanine transaminase, albumin, total bilirubin and alkaline phosphatase.

**Table S4 Adjusted* odds ratio (ORs) and 95% confidence intervals (95% CIs) for osteoporosis associated with lipid biomarkers (per standard deviation increase) in patients with chronic disorders**

| Independent Variable | *P* | OR (95% CI) |
| --- | --- | --- |
| Total cholesterol | 0.049 | 1.42 (1.00-2.01) |
| Low-density Lipoprotein cholesterol | 0.189 | 1.25 (0.90-1.73) |
| High-density Lipoprotein cholesterol | 0.647 | 1.09 (0.76-1.54) |
| Triglyceride | 0.008 | 1.66 (1.14-2.41) |

*Adjusted for sex, age, body mass index, type 2 diabetes, neuropathy, biguanide, calcium channel blockers, angiotensin converting enzyme inhibitors, alanine transaminase, albumin, total bilirubin and alkaline phosphatase.
